# Characterization of the Genetic Diversity of Acid Lime (*Citrus aurantifolia* (Christm.) Swingle) Cultivars of Eastern Nepal Using Inter-Simple Sequence Repeat Markers

**DOI:** 10.3390/plants7020046

**Published:** 2018-06-12

**Authors:** Nabin Narayan Munankarmi, Neesha Rana, Tribikram Bhattarai, Ram Lal Shrestha, Bal Krishna Joshi, Bikash Baral, Sangita Shrestha

**Affiliations:** 1 Central Department of Biotechnology, Tribhuvan University (CDBt-TU), GPO Box 44613, Kirtipur, Kathmandu, Nepal; tribikrambhattarai@gmail.com; 2 Molecular Biotechnology Unit, Faculty of Science, Nepal Academy of Science and Technology (NAST), GPO Box 3323, Khumaltar, Lalitpur, Nepal; neesarana@hotmail.com (N.R.); sangitamaleku@gmail.com (S.S.); 3 Nepal Agriculture Research Council (NARC), GPO Box 5459, Khumaltar, Lalitpur, Nepal; rals_135@yahoo.com (R.L.S.); joshibalak@yahoo.com (B.K.J.); 4 Department of Biochemistry, University of Turku, FIN-20014 Turku, Finland

**Keywords:** citrus breeding, diversity, genetic similarity, lime, molecular markers, PCR

## Abstract

Acid lime (*Citrus aurantifolia* (Christm.) Swingle) is an important fruit crop, which has high commercial value and is cultivated in 60 out of the 77 districts representing all geographical landscapes of Nepal. A lack of improved high-yielding varieties, infestation with various diseases, and pests, as well as poor management practices might have contributed to its extremely reduced productivity, which necessitates a reliable understanding of genetic diversity in existing cultivars. Hereby, we aim to characterize the genetic diversity of acid lime cultivars cultivated at three different agro-ecological gradients of eastern Nepal, employing PCR-based inter-simple sequence repeat (ISSR) markers. Altogether, 21 polymorphic ISSR markers were used to assess the genetic diversity in 60 acid lime cultivars sampled from different geographical locations. Analysis of binary data matrix was performed on the basis of bands obtained, and principal coordinate analysis and phenogram construction were performed using different computer algorithms. ISSR profiling yielded 234 amplicons, of which 87.18% were polymorphic. The number of amplified fragments ranged from 7–18, with amplicon size ranging from ca. 250–3200 bp. The Numerical Taxonomy and Multivariate System (NTSYS)-based cluster analysis using the unweighted pair group method of arithmetic averages (UPGMA) algorithm and Dice similarity coefficient separated 60 cultivars into two major and three minor clusters. Genetic diversity analysis using Popgene ver. 1.32 revealed the highest percentage of polymorphic bands (PPB), Nei’s genetic diversity (H), and Shannon’s information index (I) for the Terai zone (PPB = 69.66%; H = 0.215; I = 0.325), and the lowest of all three for the high hill zone (PPB = 55.13%; H = 0.173; I = 0.262). Thus, our data indicate that the ISSR marker has been successfully employed for evaluating the genetic diversity of Nepalese acid lime cultivars and has furnished valuable information on intrinsic genetic diversity and the relationship between cultivars that might be useful in acid lime breeding and conservation programs in Nepal.

## 1. Introduction

Citrus, an important genus from Rutaceae family [1], is an ancient perennial crop more often cultivated in tropical and sub-tropical parts of the globe [2]. Nepal is one of the countries in Asia where citrus is thought to have originated [3], and plays a crucial role in the horticultural industry. The diploid citrus plants (2*n* = 2*x* = 18) are naturally hybridized through cross-pollination, generating hybrids and also increasing the ploidy levels [4,5].

Acid lime (*Citrus aurantifolia* (Christm.) Swingle) and lemon (*Citrus limon* (L.) Burm. f.) are important fruit crops in Asia, and India is the largest producer of lime and lemon [6]. Acid lime, commonly known as “Kagati” in Nepal, is enriched in vitamin C, with multiple uses, such as for preparing juice, pickles, and salad. Its medicinal properties are attributed to its preventive and curative measures against various diseases of the joints and bones, cold, influenza, dysentery, piles, scurvy, cold, and constipation [7]. As lemon and lime juices have profound amounts of citric acid, comprising 1.38 and 1.44 g/oz, respectively [8], beverages with citric acid are reported to reduce the content of calcium, and ultimately enhancing an excretion of urinary citrates. Hence, it could be a good dietary supplement for preventing and managing calcium urolithiasis (kidney stone) [7,9].

Acid lime proves to be a commercially crucial fruit crop, cultivated in Nepal, ranking third after mandarin and sweet orange in terms of area coverage (2731 ha). The cultivation of acid lime is practiced in several districts of Nepal (60 out of 77), from low land on the outer foothills of Himalayas, known as Terai, to the lands on the high hill landscapes of Nepal, particularly concentrated in Eastern Nepal [10]. Unlike mandarins and sweet oranges, acid lime can be successfully raised from the high hill to the Terai landscapes of Nepal [10]. Out of three different varieties of lime grown commercially in Nepal (acid lime, Eureka, and hybrids), acid lime bears enormous commercial value due to its size, better aroma, and enriched medicinal properties [11]. The favorable season for the production of lime in Nepal is from September to November; however, the demand for this fruit is throughout the year [12].

In Nepal, the production of Acid lime is 8.3 ton/ha [13], which is very low compared to the productivity of other countries like Argentina (19 ton/ha) and India (12.2 ton/ha) [14]. The reason for this low production may be due to several stressors, like biotic (pests and diseases) and abiotic stress (salinity, drought, and temperature). In addition, prevailing climate change is putting more pressure on the gross crop productivity [15]. Considering these scenarios, elite acid lime varieties with desirable qualities—such as nematode resistance, resistance to disease, juice content, higher yield, stress tolerance, etc.—hold great promise. Development of such cultivars with desirable qualities can be achieved via breeding programs (conventional and non-conventional) viz., molecular marker-assisted breeding, protoplast fusion, mutation breeding, and genetic engineering [16,17,18]. Therefore, the study of genetic diversity at the molecular level would furnish valuable information regarding the diversity in the gene pool, which could be harnessed for a breeding and cultivar development program as well as for the conservation of acid lime germplasm in Nepal.

The major motive of plant breeders lies in improving the qualitative and quantitative traits of the existing cultivars, which has been achieved via conventional breeding that involves whole genomes followed by the selection of highest quality recombinants among several segregating individuals. However, this is highly time-consuming and tedious, because it requires multiple crosses and several generations, vigilant linkage drag, and phenotypic selection [19]. Recently developed DNA-based molecular marker technologies have offered several options to plant breeders as complementary tools to conventional breeding for the improvement of crops, such as the assessment of genetic diversity, identification of cultivars, marker-assisted selection and breeding, and more recently, genomics-assisted breeding [20,21,22,23,24].

Three different classes of molecular markers are currently available for plant breeders to expedite crop improvement. These include (i) hybridization-based markers, such as restriction fragment length polymorphisms (RFLPs); (ii) PCR-based markers, such as amplification fragment-length polymorphisms (AFLPs), microsatellite, or simple sequence repeats (SSRs); and (iii) sequencing-based, such as single nucleotide polymorphisms (SNPs) [19]. These molecular markers prove useful for various purposes in crop improvement programs, such as (i) for the construction of saturated molecular genetic linkage maps (physical and genetic) in various species [25,26]; (ii) for identification of markers associated with genes/quantitative trait loci (QTL) that control traits of economic importance for indirect marker-assisted selection (MAS); (iii) gene introgression through backcrossing; (iv) germplasm characterization, genetic diversity assessment, and cultivar identification; and (v) genome organization and phylogenetics, etc. [27,28,29].

Of the various molecular-marker systems, the PCR-based ISSR marker system has wide usage in studying genetics [30,31,32]. These markers are polymorphic in nature [33], abundant in the genome [34], and have the advantages of SSR markers, circumventing the major obstacle of the development of SSR markers (i.e., the requirement of flanking sequences for primer design) and enjoying the advantages of random markers [35]. ISSR technique combines the benefits of AFLP and SSR markers with Random Amplified Polymorphic DNA (RAPD) marker’s universality [21]. The ISSR markers are informative for species where genome sequences are unavailable [36], and has a capacity to rapidly screen and differentiate between closely related individuals [30]. The major drawback of ISSR markers is that they are dominant and inherited in the Mendelian fashion [37,38,39], so they may influence genetic diversity estimation.

Several molecular marker-based studies like RAPD and ISSR have been conducted in different citrus germplasms [23,40,41,42,43]. Although the co-dominant SSR marker system-based genetic study was carried out prior to this study, using the same acid lime samples [41], it was not based on advanced capillary electrophoresis (CE), and was carried out using the conventional agarose electrophoretic system. An overall objective of this research was to furnish a baseline data to assist with breeding and conservation programs for acid lime in Nepal. Our main research questions were: (i) what is the extent of genetic diversity in the acid lime cultivars of Eastern Nepal, and (ii) which agro-ecological zone harbors the most genetically diverse acid lime cultivars based on our present ISSR study? We anticipate that the results obtained from this study would be valuable for current and future acid lime breeding programs, as well as Agro-biodiversity conservationists of Nepal. Therefore, our objectives were to employ an ISSR marker system to evaluate the genetic diversity of the same acid lime germplasm sampled from different agro-ecological zones of Eastern Nepal that were used previously with two other two marker systems, viz., RAPDs and SSRs, and to perform a comparative discussion based on the results obtained from all three different marker systems: RAPD, SSRs, and ISSRs [41,43].

## 2. Material and Methods

### 2.1. Plant Materials

**Figure 1:**
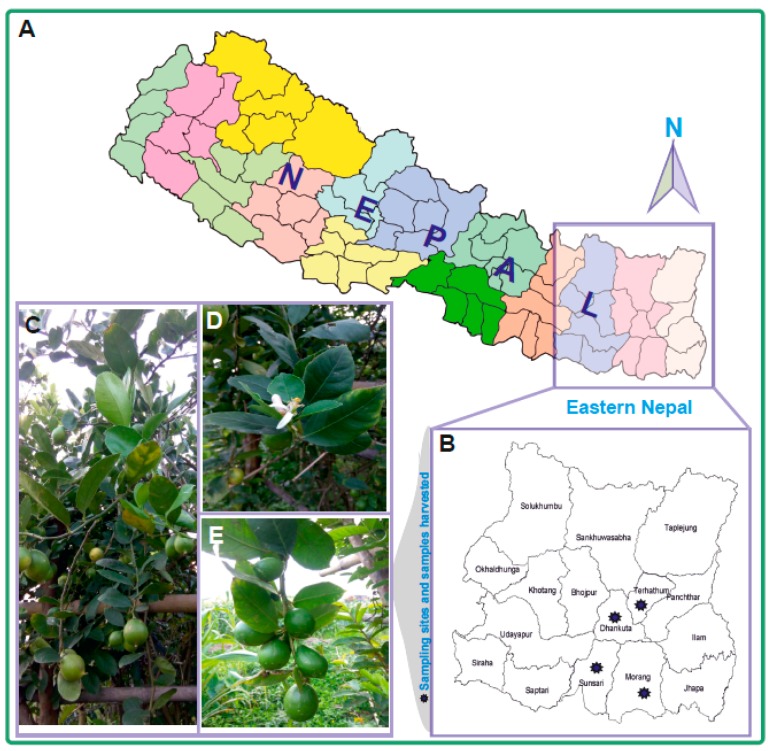
The sampling sites and acid lime pictures used for the experiment. (**A**) Map of Nepal; (**B**) sites from where the samples were harvested; (**C**–**E**) trees and branches from which the samples were harvested.

Altogether, 60 young and healthy leaves from 60 acid lime tree samples (6 to 8 weeks old) were harvested from farmer’s plantation areas representing three agro-ecological zones of Eastern Nepal (20, 21, and 19 samples from low land Terai, mid-hill, and high hill zones, respectively) and were stored in airtight zip-lock bags with silica gel (Figure 1). Trees were randomly selected from all agro-ecological zones, viz., Terai, mid-hill, and high hill (Table 1).

### 2.2. DNA Extraction and PCR Amplification

Dried leaf samples (100 mg) were ground to a fine powder using liquid nitrogen, and the genomic DNA was extracted according to the protocol of DNeasy plant DNA extraction mini-kit (QIAGEN, Inc., Valencia, CA, USA). ISSR–PCR amplification was performed in 25 µL total reaction volume having 25 ng of genomic DNA, 3.0 mM MgCl_2_, 2.5 µL (10 mM) of 10× PCR reaction buffer (Thermo Fisher Scientific, Waltham, MA, USA), 0.4 µM primer, 0.4 mM dNTPs, and 1.5 U *Taq* polymerase (Thermo Fisher Scientific, Waltham, MA, USA). The PCR cycling conditions consisted of initial denaturation of 94 °C (2 min) followed by 40 cycles of denaturation at 94 °C (30 s), annealing at 50 °C (45 s), elongation at 72 °C (2 min), and a final elongation at 72 °C (7 min), followed by a hold at 4 °C (for infinity) [23].

The PCR products obtained were analyzed in 2% (*w*/*v*) agarose gel comprised of Ethidium Bromide (0.5 μg/mL, Promega Co., Madison, WI, USA) [44] after running the PCR in 1× TAE Buffer (50 V; 2 h). A Gel-doc system (Ingenius, Syngene Bioimaging, London, UK) was employed for gel visualization and documentation of the ISSR bands. The size of the PCR products was determined by using two DNA ladders, viz., Gene Ruler 100 bp Plus DNA ladder (Catalog number SM0323, Thermo Fisher Scientific, Waltham, MA, USA) and AccuLadder 100 bp DNA size marker (Catalog number D-1030-1, Bioneer, Alameda, CA, USA).

### 2.3. Inter-Simple Sequence Repeat Profiling and Scoring of the Data

Using optimized ISSR–PCR reaction conditions (25 ng genomic DNA, 3.0 mM MgCl_2_, 2.5 µL (10 mM), 10× PCR reaction buffer (Thermo Fisher Scientific, Waltham, MA, USA), 0.4 µM Primer, 0.4 mM dNTPs, and 1.5 U *Taq* polymerase in a 25 µL reaction volume) and cycling conditions, as mentioned in the previous section (Section 2.2), 49 different ISSR oligos were used to screen the acid lime genomic DNA samples. All profiling experiments were repeated twice to check the reproducibility of PCR amplifications. Out of 49 oligos suggested by different researchers in their published papers [23,45,46], 21 oligos that provided crispy, multiple, scorable, and reproducible bands with our samples were selected for further ISSR profiling. The ISSR profiles generated by each of the 21 oligos were used to score the bands and for the creation of binary data matrix. Scoring of all polymorphic and monomorphic bands was performed. Scoring of the markers as 0, 1, or 9 was performed to indicate absence, presence, or failure of the PCR amplification (250–3200 bp) respectively [47,48,49].

### 2.4. Data Analysis

The binary data matrix was analyzed using Microsoft Excel 2007. The matrix estimates the banding characteristics, such as (i) the total number of bands obtained (TNB), (ii) the number of polymorphic bands (NPB), (iii) percentage polymorphism (PP), (iv) polymorphic information content (PIC), (v) band informativeness (I_B_), and (vi) resolving power (R_P_) for each primer used: {PP = NPB/TNB generated by each primer; PIC = 1 − ∑(P_ij_)^2^, where P_ij_ is the frequency of the *i*-th pattern revealed by the *j*-th primer, summed across all patterns revealed by the primers [50]; I_B_ = 1 − [2 × (0.5 − *p*)], where *p* represents the proportion of cultivars comprising the band; R*_p_* = ∑I_B_ [51]}.

The genetic diversity in acid lime was computed using Numerical Taxonomy and Multivariate System (NTSYS, version 2.21i, New York, NY, USA). Similarity indices were computed by applying a similarity to the qualitative data. From these similarity indices, sequential, agglomerative, hierarchical, and nested (SAHN) clustering was performed using the unweighted pair group method of arithmetic averages (UPGMA) algorithm [52]. Similarity coefficients were computed based on three different measures: simple matching coefficient (SM) [53], Dice’s coefficient of similarity (D) [47,54], and Jaccard’s coefficient (J) [48]. The matrices of the SM, J, and D coefficients were compared by a Mantel test [55] using the MXCOMP (Matrix Comparison) option in NTSYS program. The cophenetic correlation test was applied for estimating the correlation between each of the similarity matrices and its corresponding phenogram. The estimated correlation coefficient values showed the goodness of fit of cluster analysis performed on the basis of each of SM, J, and D. In order to evaluate the tree generated from the UPGMA clustering by genetic similarity coefficients, consensus fork indices (CI_C_) were calculated using the strict consensus method of the NTSYS program for each combination of similarity coefficient and UPGMA clustering. CI_c_ measures how resolved the tree is [51]. The best-fitted similarity matrix coefficient was then employed for the assessment of the genetic diversity.

In addition, AMOVA (analysis of molecular variance) was performed among and within three agro-ecological zones. The genetic diversity and relationship between the acid lime cultivars of these different agro-ecological zones of Eastern Nepal were also studied through a principal coordinate analysis (PCoA), using the Multivariate Statistical Package (MVSP, version 3.21), and in terms of percentage of polymorphic bands (PPB), Nei’s genetic diversity (H), and Shannon’s information index (I) using Popgene (version 1.32).

## 3. Results

### 3.1. Inter-Simple Sequence Repeat Polymorphism in Acid Lime Cultivars

**Figure 2:**
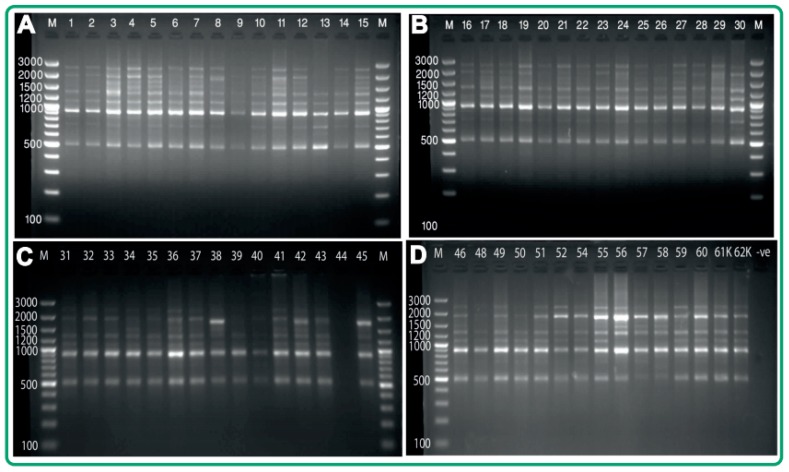
Inter-simple sequence repeat (ISSR) profile generated by the primer (C8). Lanes marked with M is 100 bp plus molecular weight markers. (**A**) Lanes 1–15 represent acid lime samples 1–15; (**B**) lanes 16–30 represent Acid lime samples 16–30; (**C**) lanes 31–45 represent acid lime samples 31–45; (**D**) lanes 46–62K represent acid lime samples 46–62K.

Altogether, 234 loci were amplified by 21 ISSR primers across the 60 acid lime cultivars (coming from three agro-ecological zones), with an average amplification of 9.72 bands per primer (Figure 2). Among the amplified bands, 204 (87.18%) were revealed to be polymorphic, and 30 (12.82%) were monomorphic. The polymorphic bands produced by different oligos ranged from 55.56–100% (eight oligos revealed 100% polymorphism). The number of scorable bands produced per primer ranged from 7–18, with variation in amplicon size ranging from 250–3200 bp. In total cultivars, the highest number of ISSR loci (18) was produced by the primer UBC 857, whereas the lowest number of ISSR loci (7) was produced by the primer C1 and UBC 834 (Supplementary Table S1).

The polymorphic information content (PIC) value ranged from 0.74 (UBC 807) to 0.93 (UBC 857) with an average of 0.85. The band informativeness (I_B_) of the 21 ISSR primers ranged from 0.42 (C4) to 1.77 (C1), with an average of 1.12, and the resolving power (R_P_) ranged from 4.63 (UBC 807) to 23.16 (UBC 857), with an average of 12.03. Here we found that the primers that have the highest PIC value also gave the highest R_P_ score (Supplementary Table S1).

AMOVA analysis revealed significant (*p* < 0.001) partitioning of the genetic variation (14% occurring among agro-ecological zones, while remaining 86% within the zone). The genetic differentiation (Φ_PT_) for the three agro-ecological zones was 0.139, suggesting that genetic differentiation of acid lime was comparatively lower than the genetic differentiation of acid lime within agro-ecological zone variation, accounting for the 86% variation (Supplementary Table S2).

### 3.2. Genetic Diversity in Acid Lime Cultivars

The varied range of similarity indices were obtained using simple matching (SM), Jaccard’s (J) and Dice’s (D) coefficient, i.e., SM (0.54–0.94), J (0.42–0.90), and D (0.57–0.95), with an average similarity coefficient value of 0.79, 0.69, and 0.81, respectively. The Mantel test (matrix comparison) result of the original matrices showed the correlation value between J and D to be the highest and most significant (0.99710), in comparison to SM and J (0.98143) or SM and D (0.98318) (Supplementary Table S3).

The highest CI_C_ value (CI_C_ = 1.00000) was observed for the J and D coefficients (Supplementary Table S4). The cophenetic correlation coefficient value (*r*) between the genetic similarity matrices and cophenetic matrices are presented in Supplementary Table S5. The unweighted pair group method of arithmetic average (UPGMA) distance for the D coefficient gave the highest cophenetic correlation value (*r* = 0.90356) (Supplementary Table S5), indicating a very good fit for the cluster analysis.

On comparative analysis made for the similarity coefficients, Dice’s coefficient was revealed to be the best, which then subjected to further interpretations of genetic diversity estimates and the relationships between various cultivars of acid lime representing different geographical gradients. Based on the Dice similarity coefficient, genetic similarity within 60 acid lime cultivars ranged from 57–95%, with an average of 81% (Supplementary Table S6). The individual genetic similarity/distance among various *C. aurantifolia* cultivars have been assessed from the pair-wise comparison of the Dice similarity matrix, which revealed the high-hill cultivars LT-20 and LT-21 to be the most genetically similar (0.95), and the Terai cultivars LS-35 and LS-56 to be the most genetically distant (0.571) genotypes. Considering these similarity indices, Terai cultivars were shown to have a wider genetic base, followed by mid-hill and high-hill cultivars.

The genetic diversity indices of acid lime cultivars from different agro-ecological zones that were assessed based on percentage of polymorphic band (PPB), Nei’s gene diversity (H), and Shannon’s information index (I), using Popgene ver. 1.32, revealed the highest diversity indices in Terai cultivars (PPB = 69.7%; H = 0.215; I = 0.33), followed by mid-hill (PPB = 66.7%; H = 0.20; I = 0.30) and high-hill (PPB = 55.1%; H = 0.17; I = 0.26) (Supplementary Table S7).

### 3.3. The Genetic Relationship Based on Unweighted Pair Group Method of Arithmetic Averages Cluster Analysis and Principal Coordinate Analysis

**Figure 3:**
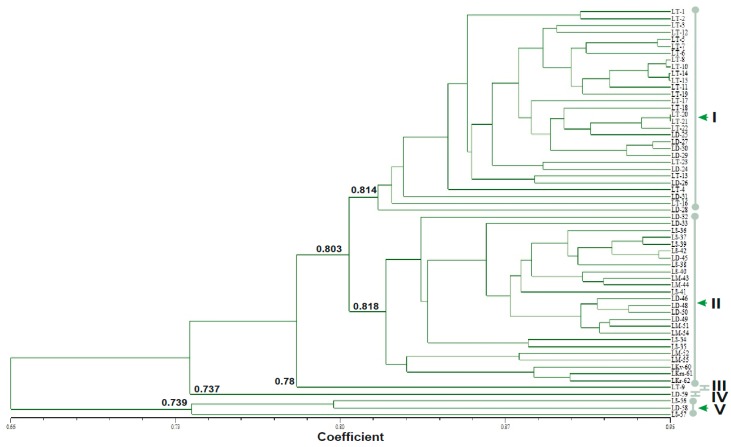
Phenogram generated for 60 *Citrus aurantifolia* (Acid lime) cultivars (please refer to Table 1 for sample details) by unweighted pair group method of arithmetic averages (UPGMA) cluster analysis, using the Dice similarity coefficient computed from 234 ISSR loci generated by 21 primers. The clusters are labeled as I, II, III, IV, and V.

The 60 cultivars of acid lime analyzed were separated into two major clusters (I and II) and three minor clusters (III, IV, and V) in the phenogram (Figure 3). The cultivars from the high-hill, mid-hill, and Terai zones were intermingled in different clusters. Cluster I was comprised of 30 cultivars from the high-hill and mid-hill agro-ecological zones. In this cluster, the highest genetic similarity coefficient was observed for cultivars LT-20 and LT-21 (0.95), and the lowest similarity coefficient (i.e., highest genetic distance) was observed between LS-35 and LS-56 (0.57). Cluster II was comprised of 25 cultivars from the high-hill, mid-hill, and Terai agro-ecological zones, along with the exotic varieties of Vanarasi, Madrasi, and Rampur (LKv-60, LKm-61, and LKr-62 respectively). In this cluster, cultivars LS-37 and LS-39 had the highest similarity value of 0.99, followed by 0.94 between LS-42 and LD-45, 0.93 between LD-48 and LD-50, and so on. Cluster III and cluster IV each consisted of a single cultivar, LT-9 and LD-59 from high-hill and mid-hill, respectively. Cluster V was comprised of three cultivars (LS-56, LS-57, and LD-58) that belong to the Terai agro-ecological zone (Supplementary Table S8). Cluster II was separated from cluster I at a similarity coefficient of 0.80, and cluster IV separated from rest of the group at a similarity coefficient of 0.66. There was little genetic variation between clusters I and II (similarity of 81.4% and 81.8%, respectively) and clusters IV and V (similarity of 73.7% and 73.9%, respectively), whereas a wider variation was observed between cluster IV and II (Figure 3).

**Figure 4:**
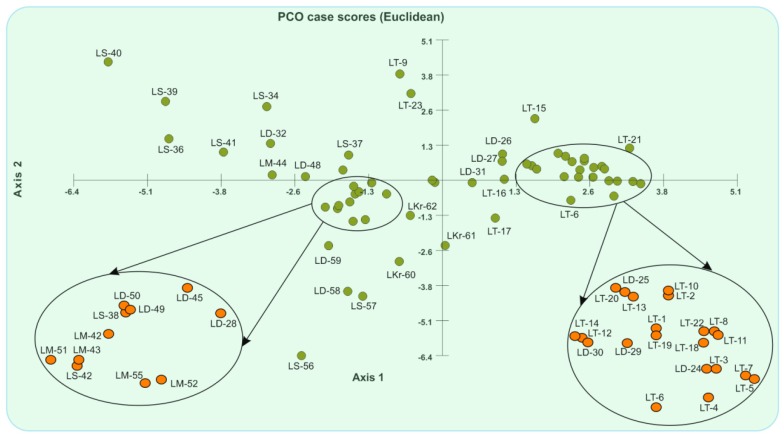
Principal coordinate analysis (PCoA) of Dice’s similarity matrix, carried out with Multivariate Statistical Package (MVSP) 3.21.

Two-dimensional plots of the principal coordinate analysis (PCoA) classified the 60 acid lime cultivars based on ISSR allelic variation (Figure 4). The first principal coordinate axis accounted for 14.51% (Eigen value = 323.27; percentage of variance = 14.51%), and the second accounted for 8.34% (Eigen value = 185.77; percentage of variance = 8.34%) of the total genetic variation, with a cumulative variation of 22.85%. Therefore, the groups were combined, with axes 1 and 2 expanding 22.85% of the total variation.

## 4. Discussion

### 4.1. Inter-Simple Sequence Repeat Polymorphism and Genetic Diversity Estimation in Nepalese Acid Lime Cultivars

Polymorphism reported in *Citrus* spp. is comparable with our present investigation (PP = 87.18%), such as 89.4% in wild *Citrus* spp. [23], 87% in *C. indica* [56], and 100% in a few commercially important *Citrus* spp. [45]. The total amplification profiles generated by the 21 ISSR primers yielded 234 bands, of which 204 were polymorphic and 30 were monomorphic, which provides us with a clue about the existence of high level of genetic diversity among randomly selected acid lime cultivars from three different agro-ecological zones of Eastern Nepal. The RAPD screening system screens the whole genome, as revealed by 94.94% polymorphism in the corresponding samples, which is much higher compared to previous findings [43]. However, ISSR amplicons correspond to the specific SSR loci, and ISSR–PCR is more stringent than RAPD, because of the use of longer oligos (16–25 bp) that allows for the use of high annealing temperatures [21]. The PIC value provides valuable information about heterozygosity and is associated with the degree of polymorphism. A primer/primer pair with comparably higher PIC values is useful in discriminating between cultivars [57]. In our study, the PIC value ranged from 0.74 to 0.93, with an average of 0.89. Interestingly, the PIC value of the SSR-based study of the same germplasm revealed a comparatively low (0.5) value [41], which is due to SSR markers being co-dominant and specific to a PCR-based marker system. Also, as the SSR-based study was conducted in a conventional agarose gel electrophoretic system (in contrast to a polyacrylamide or capillary electrophoresis), small allele size differences (varying in few bases) might not have been properly resolved. Capillary electrophoresis has been shown to be the superior technique for SSR-based genetic analysis [58]. However, the PIC value of the present study is comparable to that reported in our previous RAPD-based study (ranged from 0.78 to 0.88 with an average of 0.80) [43]. Our current investigation revealed the primer (UBC 857) with the highest PIC value (0.93) to have highest resolving power value (23.16) (Supplementary Table S2), which provided a glimpse of quantitative data and allowed us to make direct comparisons between the primers [51].

The clustering based on UPGMA analysis revealed the genetic diversity and relationship among acid lime cultivars of three geographically diverse agro-ecological zones. No specific cluster was formed for cultivars from the different agro-ecological zones under study. Our result is congruent with SSR-based findings as being separated into two major and three minor clusters, apart from the distribution of different cultivars in different clusters [41]. Cluster I of the present study was comprised of cultivars from the high- and mid-hill zones, revealing the close genetic relationship, whereas cluster II comprised of cultivars from all three agro-ecological zones and the exotic varieties, viz., Vanarasi, Madrasi, and Rampur (LKv-60, LKm-61, and LKr-62, respectively). These exotic varieties were also clustered together on the phenogram generated from the SSR [41] and RAPD markers [43]. Cultivars LS-56, LS-57, and LD-58 from the Terai ecological zone are clustered together, similar to the results obtained in the SSR-based phenogram by Shrestha et al., (2012a), indicating their genetic closeness. The intermixing of cultivars that are grown in different agro-ecological domains in different clusters in the phenogram may be attributed to the genetic similarities among different cultivars of various qualitative and quantitative traits. In order to improve varieties, the ideal parent for hybridization should be selected based on the level of genetic diversity estimated using molecular markers [59]. The high usage of morphological traits for the determination of a genetic relationship among plants and its varieties exists [60]. However, morphological markers do not often reflect genetic relationships, because of their interaction with the environment and epistasis [61]. On the basis of a previous study on fruit diversity and vitamin C content, four elite cultivars (two from the high hills and one each from Terai and the mid-hills) were confirmed to be of superior quality and recommended for conservation, breeding, and various developmental purposes [62]. In our present investigation, the first two cultivars (LT-17 and LT-23) are clustered together in group I, and the remaining two (LD-49 and LM-44) are clustered in group II.

Dice’s similarity matrix-based genetic diversity estimates within each of three agro-ecological zones revealed a wide genetic base in cultivars from the Terai agro-ecological zone (0.57–0.94) in comparison with the mid-hill (0.70–0.94%) and high-hill (0.75–0.95%) zones. However, regarding the genetic base between agro-ecological zones, the highest value was observed between the high-hill and Terai cultivars (57–95%), followed by mid-hill and Terai cultivars (57–94%) (Supplementary Table S6). The Terai cultivars, being most genetically diverse, had a comparable genetic base to those of the mid-hill versus Terai and high-hill versus Terai. The result is comparable with the result obtained using SSR markers, where cultivars from high- and mid-hill zones have higher average genetic similarities (73% and 81%) in comparison to those from the Terai zone (69%) [41]. Our results showed that the collected cultivars from different agro-ecological zones were not genetically distinct but do have highest diversity in Terai cultivars compared to those from the high- and mid-hill areas. In our investigation, diversity indices like Shannon’s information index (I) and Nei’s gene diversity (H) were found to be 0.325 and 0.215, respectively, in the Terai agro-ecological zone, which was highest among the three zones studied. This indicates that Terai has a diverse gene pool, compared to mid- and high-hill regions. This discrepancy in the diverse gene pool could be due to the higher accessibility of movement for the germplasm within the country in Terai, and also from neighboring country India. In contrast, the lower level of genetic variability observed in the mid- and high-hill regions might be due to acid lime trees being established in natural conditions in these zones [41]. Our previous study using same germplasm based on RAPD markers, showed similar diversity indices values [43].

### 4.2. Use of Inter-Simple Sequence Repeat-Based Genetic Diversity Estimates in an Acid Lime Breeding Program in Nepal

Many qualitative and quantitative agronomic traits, such as high juice content, fruit size, and disease and insect resistance have a genetic basis of inheritance and can be enhanced by the use of molecular markers and marker-assisted selection (MAS) techniques. Selection and improvement of good qualitative and quantitative traits are important steps in the variety of developmental programs. Moreover, breeding of good quality traits requires selection of parents with greater genetic diversity [63]. For this, sufficient knowledge about genetic diversity in the gene pool is required, in order to adopt an efficient and valuable breeding approach. The present investigation has furnished genetic diversity estimates in the acid lime cultivars of eastern Nepal.

Using 60 cultivars of acid lime from three different agro-ecological zones, we investigated the genetic diversity of Nepalese acid lime using ISSR markers. Moderately high genetic polymorphism (87.18%) was detected using 21 primers. The cluster analysis revealed heterogeneous grouping into two major and three minor clusters; however, no clustering based on geographical locations was evident. This high genetic polymorphism indicates that these cultivars might also harbor diverse, agronomically important qualitative and quantitative traits, such as disease and pest resistance, physico-chemical properties, and yield. In-depth studies on these various aspects are still lacking in Nepal. However, a study on physicochemical properties of the same acid lime cultivars has been conducted [60]. Four acid lime elite cultivars (viz., LD-49 from the mid-hill zone, belonging to cluster II; LT-17 and LT-23 from the high-hill zone, belonging to cluster I; and LM-44 from the Terai zone, belonging to cluster II) were selected from this study, on the basis of the fruits’ nutritional composition and their characters such as vitamin C content, total soluble solids, amount of juice content, and titrable acidity. These cultivars can now be used as breeding material or genetic stocks for the future breeding program and genetic distance between these and others can be used as parental selection for breeding.

## 5. Conclusions

ISSR markers have been extensively used as important DNA-based molecular marker tools for the genetic diversity characterization of germplasm and the establishment of the identity of varieties, hybrids, and parental sources in plant breeding, marker trait association, germplasm management, etc. ISSR markers have also been shown to be associated with various agronomically important traits (such as disease resistance) in various crops. Our study revealed the existence of moderately high genetic diversity in acid lime cultivars of Eastern Nepal, indicating availability of various sources of acid lime genes to be exploited in current and future breeding programs. Further research needs to be undertaken to exploit ISSR and other marker systems to generate agronomically important gene- and trait-specific markers and their subsequent utilization in acid lime breeding program.

## Figures and Tables

**Table 1:** Altitudinal range, cultivar numbers, and locality details of the acid lime samples.

Above 1200 m asL			600–1200 m asL			Less Than 600 m asL		
Cult. No.	Altitude (m asL)	VDC-Ward No.	Cult. No.	Altitude, (m asL)	VDC-Ward No.	Cult. No.	Altitude, (m asL)	VDC-Ward No.
LD-24	1290	Balehara-8	LD-25	1180	Balara-1	LD-45	135	Sunpur-2
LD-46	1278	Bodhe-2	LD-26	1175	Balara-1	LD-58	135	Sunpur-2
LD-50	1638	Rajarani-9	LD-27	1175	Balara-1	LM-43	135	Sunpur-2
LT-1	1605	Okhre-8	LD-28	1175	Balara-1	LM-44	135	Sunpur-2
LT-2	1285	Okhre-1	LD-29	1175	Balara-1	LM-51	125	Pathari-2
LT-3	1305	Okhre-8	LD-30	1175	Balara-1	LM-52	125	Pathari-2
LT-8	1505	Okhre-8	LD-31	1150	Dhnk-3	LM-54	125	Pathari-2
LT-9	1500	Okhre-5	LD-32	1130	Balhra-3	LM-55	125	Pathari-2
LT-12	1310	Fachamara-7	LD-33	1130	Balhra-1	LS-34	128	Narsing-2
LT-13	1315	Fachamara-7	LD-48	1181	Bodhe-1	LS-35	128	Narsing-4
LT-14	1308	Fachamara-7	LD-49	1185	Bodhe-1	LS-36	128	Narsing-4
LT-15	1655	Fachmara-9	LD-59	1175	Balara-1	LS-37	128	Narsing-4
LT-16	1405	Fachamara-7	LKm-61	1285	Balara-1	LS-38	128	Narsing-4
LT-17	1750	Fachmara-7	LKr-62	1285	Balara-1	LS-39	128	Narsing-4
LT-18	1710	Fachmara-9	LKv-60	1285	Balara-1	LS-40	128	Narsing-4
LT-19	1350	Fachamara-7	LT-4	1155	Okhre-1	LS-41	128	Narsing-4
LT-20	1410	Fachamara-8	LT-5	1155	Okhre-3	LS-42	128	Narsing-4
LT-21	1485	Fachamara-1	LT-6	1150	Okhre-3	LS-56	128	Narsing-4
LT-22	1505	Sudap-1	LT-7	1145	Okhre-2	LS-57	128	Narsing-4
LT-23	1308	Sudap-7	LT-10	1135	Okhre-3			
			LT-11	1130	Okhre-3			

Cult. no.: Cultivar number; LD: Lime Dhankuta district; LKm: Lime Madras; LKr: Lime Rampur; LKv: Lime Varanasi; LM: Lime Morang district; LS: Lime Sunsari district; LT; Lime Terhathum district; VDC: Village Development Committee; m: meter; asL: above sea level.

## Supplementary Materials

The following are available online at http://www.mdpi.com/2223-7747/7/2/46/s1; Table S1: ISSR primer details, Total Number of amplified Bands (TNB), Number of Polymorphic Bands (NPB), Percent Polymorphism (PP), amplicon size range, Polymorphic Information Content (PIC), Band Informativeness (I_B_) and Resolving power (R_P_) computed for different ISSR primers used to generate ISSR profiles in 60 *Citrus aurantifolia* (Acid lime) accessions; Table S2: Analysis of Molecular Variance (AMOVA) for 60 Acid lime accessions from three agro-ecological regions; Table S3: Correlation Coefficient values generated from Mantel test of original similarity matrices; Table S4: Consensus Fork Index (CI_C_) values generated for the UPGMA based phenogram using different similarity coefficients; Table S5: Cophenetic correlation coefficients value (r) obtained for different similarity matrices *viz*. Simple Matching, Jaccard’s and Dice; Table S6: Percentage genetic similarities observed within and between three agro-ecological zones for various Acid lime samples based on Dice similarity matrix generated from ISSR profile; Table S7: Genetic variation observed for *C. aurantifolia* samples representing different agro-ecological zones as revealed by POPGENE ver. 1.32; Table S8: Acid lime accessions belonging to two major and three minor clusters of UPGMA phenogram.
