# Supplementary material for: Characterization of the Genetic Diversity of Acid Lime (Citrus aurantifolia (Christm.) Swingle) Cultivars of Eastern Nepal Using Inter-Simple Sequence Repeat Markers
Source: Plants (Basel). 2018 Jun 12;7(2):46. doi: 10.3390/plants7020046 (PMC6027463; doi:10.3390/plants7020046)
Supplement: Supplementary file 1 [file plants-07-00046-s001.pdf]

Characterization of the Genetic Diversity of Acid Lime (*Citrus aurantifolia* (Christm.) Swingle) Cultivars of Eastern Nepal Using Inter-Simple Sequence Repeat Markers

Nabin Narayan Munankarmi, Neesha Rana, Tribikram Bhattarai, Ram Lal Shrestha, Bal Krishna Joshi, Bikash Baral, Sangita Shrestha

Table S1. ISSR primer details, Total Number of amplified Bands (TNB), Number of Polymorphic Bands (NPB), Percent Polymorphism (PP), amplicon size range, Polymorphic Information Content (PIC), Band Informativeness (I_B) and Resolving power (R_P) computed for different ISSR primers used to generate ISSR profiles in 60 *Citrus aurantifolia* (Acid lime) accessions.

S.N.	Primer Code	Primer Sequence (5′ – 3′)	Primer Length (bp)	TNB	NPB	PP	Amplicon size range (bp)	PIC	I_B	R_P
1	C1	TCTCTCTCTCTCTCTCTCCC	20	7	5	71.43	550-1800	0.85	1.77	12.40
2	C2	AGCAGCAGCAGCGT	14	10	10	100	500-3200	0.83	0.83	8.33
3	C4	CTCCTCCTCGC	11	14	14	100	300-2200	0.82	0.42	5.87
4	C5	CACCACCACGC	11	12	11	91.67	600-2500	0.83	0.60	7.23
5	C7	HVHGAGAGAGAGAGAGAT	18	17	15	88.23	250-2000	0.89	0.87	14.87
6	C8	TCCTCCTCCTCCTCCRY	17	9	7	77.78	520-2800	0.87	1.47	13.23
7	C9	BDBTCCTCCTCCTCCTCC	18	9	6	66.67	520-2000	0.86	1.50	13.56
8	C10	HVHTCCTCCTCCTCCTCC	18	11	8	72.73	500-2000	0.79	0.78	8.63
9	UBC 807	AGAGAGAGAGAGAGAGT	17	9	9	100	450-1300	0.74	0.51	4.63
10	UBC 810	GAGAGAGAGAGAGAGAT	17	16	16	100	390-1980	0.85	1.00	12.00

11	UBC 812	GAGAGAGAGAGAGAGAA	17	12	9	75.00	450-1500	0.91	1.03	16.60
12	UBC 825	ACACACACACACACACT	17	9	5	55.56	500-2000	0.87	1.51	13.63
13	UBC 834	AGAGAGAGAGAGAGAGYT	18	7	5	71.43	310-1550	0.81	1.28	8.96
14	UBC 835	AGAGAGAGAGAGAGAGYC	18	10	10	100	400-2900	0.86	1.29	12.96
15	UBC 836	AGAGAGAGAGAGAGAGYA	18	10	10	100	320-1450	0.87	1.13	11.30
16	UBC 841	GAGAGAGAGAGAGAGAYC	18	10	10	100	320-1700	0.87	1.08	10.83
17	UBC 842	GAGAGAGAGAGAGAGAYG	18	12	12	100	320-1650	0.89	1.04	12.53
18	UBC 857	ACACACACACACACACYG	18	18	17	94.44	300-3000	0.93	1.28	23.16
19	UBC 873	GACAGACAGACAGACA	16	14	13	92.86	470-3000	0.89	0.96	13.43
20	UBC 888	BDBCACACACACAGACA	17	9	7	77.78	480-1450	0.87	1.47	13.23
21	UBC 889	DBDACACACACACACACA	18	9	5	55.56	480-1400	0.87	1.70	15.33
	**Total**			**234**	**204**		**Average**	**0.85**	**1.12**	**12.03**
**Average Polymorphic Bands per primer = 9.72**					**Average Polymorphism = 87.18**					

H, non-G; Y, Pyrimidine; B, non-A; D, non-C; V, non-T

Table S2. Analysis of Molecular Variance (AMOVA) for 60 Acid lime accessions from three agro-ecological regions.

Source of variation	df	SS	MS	Estimated variance	Total variance	P-value	ϕ_{PT}
Among zones	2	185.227	92.613	3.539	14%	< 0.001	0.139
Within zones	57	1248.298	21.900	21.900	86%		
Total	59	1433.525		25.439	100%		

Df: Degree of freedom; SS: Sum of square; MS: Mean sum of square; ϕ_{PT}: Indicator of the genetic differentiation

Table S3. Correlation Coefficient values generated from Mantel test of original similarity matrices.

	Simple Matching (SM)	Jaccard (J)	Dice (D)
Simple Matching (SM)	******	0.98143	0.98318
Jaccard (J)		******	0.99710
Dice (D)			******

Table S4. Consensus Fork Index (CI_C) values generated for the UPGMA based phenograms using different similarity coefficients.

	Simple Matching (SM)	Jaccard (J)	Dice (D)
Simple Matching (SM)	******	0.74138	0.74138
Jaccard (J)		******	1.00000
Dice (D)			******

Table S5. Cophenetic correlation coefficients value (r) obtained for different similarity matrices *viz*. Simple Matching, Jaccard's and Dice.

Clustering module of similarity	Simple Matching	Jaccard	Dice
UPGMA	0.88396	0.89800	0.90356

Table S6. Percentage genetic similarities observed within and between three agro-ecological zones for various Acid lime samples based on Dice similarity matrix generated from ISSR profile.

Zone/Zone	High-hill	Average	Mid-hill	Average	Terai	Average
High-hill	75-95	86.02	68-95	83.6	57-95	82.68
Mid-hill			70-94	82.94	57-94	79.98
Terai					57-94	79.33

Zone: Agro-ecological zone

Table S7. Genetic variation observed for *C. aurantifolia* samples representing different agro-ecological zones as revealed by POPGENE ver. 1.32.

Population of *Citrus aurantifolia*	Sample Size	Number of polymorphic bands	PPB (%)	H	I
High-hill	20	129.00	55.130	0.173	0.262
Mid-hill	21	156.00	66.670	0.202	0.308
Terai	19	163.00	69.660	0.215	0.325
Average		149.33	63.820	0.197	0.300
Species level (Multipopulation)	60	204	87.180	0.223	0.348

PPB = Percent Polymorphic Bands/loci; H = Nei's gene diversity; I = Shannon's information index

Table S8. Acid lime accessions belonging to two major and three minor clusters of UPGMA phenogram.

S.N.	Clusters	Accessions
1	I	LT-1, LT-2, LT-3, LT-12, LT-8, LT-10, LT-14, LT-15, LT-11, LT-19, LT-5, LT-7, LT-6 , LT-17, LT-18, LT-20, LT-21, LT-22, LD-25, LD-27, LD-30, LD-29, LT-23, LD-24, LT-13, LD-26, LT-4, LD-31, LT-16, LD-28
2	II	LD-32, LD-33, LS-36, LS-37, LS-39, LS-42, LD-45, LS-38, LS-40, LM-43, LM-44, LS-41, LD-45, LD-48, LD-50, LD-49, LM-51, LM-54, LS-34, LS-35, LM-52, LM-55, LKv-60, LKm-61, LKr-62
3	III	LT-9
4	IV	LD-59
5	V	LS-56, LS-57, LD-58
